# Disentangling the Complex Relationships Between Autoimmune Diseases and Cancer Through Polygenic Risk Scores: Evidence from a Large Prospective Study

**DOI:** 10.21203/rs.3.rs-6491161/v1

**Published:** 2025-05-07

**Authors:** Xiang Shu, Jing Sun, meng zhang, Xinxuan Li, Nan Yang, Xiaohui Sun, Xinjun Wang, Xingyi Guo, Matthew Buas, Gordon Watt, Xue Li

**Affiliations:** Memorial Sloan Kettering Cancer Center; Zhejiang University School of Medicine; Zhejiang University School of Medicine; Zhejiang University School of Medicine; Zhejiang University; Zhejiang Chinese Medical University; Memorial Sloan Kettering Cancer Center; Vanderbilt University Medical Center; Memorial Sloan Kettering Cancer Center; The Netherlands Cancer Institute; School of Public Health, the Second Affiliated Hospital, Zhejiang University School of Medicine, Hangzhou, China

**Keywords:** Autoimmune diseases, Cancer risk, Cross-cancer effect, Mediation, Polygenic risk score

## Abstract

Although prior studies have reported a link between autoimmunity and carcinogenesis, the roles of genetic factors and potential mediators involved in this process remain elusive. We constructed disease-specific and combined polygenic risk scores (PRSs) for 6 common autoimmune diseases (AIDs) (systemic lupus erythematosus [SLE], rheumatoid arthritis, multiple sclerosis, Crohn’s disease, ulcerative colitis [UC], and type 1 diabetes mellitus [T1D]) in the UK Biobank cohort. Compared to those without AID, AID patients at baseline had increased risks of overall cancer, hematological, digestive, and urinary cancer. The combined AID-PRS was significantly associated with increased risks of hematological cancer (HR [95% CI] per SD increase: 1.06 [1.03–1.09]) and non-Hodgkin’s lymphoma (HR [95% CI] per SD increase: 1.10 [1.05–1.14]). For individual AID-PRSs, we identified 21 significant associations between 5 PRSs and 11 types of cancer in the overall population, along with 15 additional associations in the sex-stratified analysis. For example, SLE-, UC-, and T1D-PRS showed complex cross-cancer effects on risks of up to 6 cancer types. These associations were generally independent of immunosuppressant drug use. Differential associations of SLE-PRS with prostate cancer risk were found by prostate cancer PRS status (*P*_interaction_<0.05). Several peripheral biomarkers, including red or white blood cell counts, platelet counts, and CRP partly mediated the PRS associations (up to 16.96%). Our study provides important insights into the role of autoimmune diseases in carcinogenesis, which also highlights opportunities for target cancer screening and prevention in potentially vulnerable populations.

## Introduction

Cancer is a leading cause of premature death worldwide^1,2^. The pivotal and intricate role of immunoregulation in carcinogenesis remains as a central topic in cancer research. During cancer progression, tumor cells escape from immune attack by adopting a series of features, such as induction of inhibitory immune checkpoint molecules or reduction of the antigen presentation machinery^3,4^. Meanwhile, cancer cells orchestrate an immunosuppressive inflammatory tumor microenvironment by hijacking immune cells, which in turn facilitates immune escape, vascular remodeling, and cancer progression^5,6^. Therefore, aberrant immune responses are recognized as an important hallmark of cancer^7,8^.

Autoimmune diseases (AIDs), encompassing a family of heterogeneous diseases, are characterized by aberrant immune responses against self-antigens and chronic inflammation. The underlying immune dysregulation, or autoimmunity, has led to the hypothesis that AID patients may have a higher incidence of cancer^9^. Previous epidemiological studies have reported significant associations with risk of overall cancer^10^ and individual cancer types including Hodgkin lymphoma^11^, non-Hodgkin lymphoma^12^, and anal cancer^13^, notably in systemic lupus erythematosus patients^14^. Other case-control and cohort studies emphasized the contribution of AID to the development of alimentary tract cancers^15^. On the contrary, meta-analyses have shown that individuals with type 1 diabetes (T1D) had a lower risk of breast cancer, and rheumatoid arthritis patients had a lower risk of both colorectal and breast cancers^16,17^. This has led to speculation that the decreased risk of malignancy in AID patients may be due to enhanced immune function^9,18^. These findings highlight the complexity of the relationship between AIDs and malignancy. Furthermore, AID patients are often treated with various drugs, including immunosuppressants, nonsteroidal anti-inflammatory drugs, and anti-tumor necrosis factor-alpha therapy, depending on the type and severity of the disease. This poses an even greater challenge in assessing their independent relationship with cancer. Consequently, conventional observational studies published previously were inherently more prone to confounding bias and reverse causality, making interpretation of results challenging.

Genetic factors play an important role in AID development, with median heritability values of approximately 60% for various diseases^19^. Genome-wide association studies (GWAS) have identified dozens to hundreds of common genetic variants associated with AID risk^20–24^. Polygenic risk score (PRS) was subsequently developed to aggregate small effects of those variants. As a useful tool for quantifying an individual’s genetic predisposition, its applications range from disease prediction to risk stratification^25^. Large biobank studies such as UK Biobank provides a unique opportunity to systematically disentangle the complexity of interrelationships between the genetic predisposition to diseases, modifiable risk factors, circulating biomarkers and cancer incidence by integrating PRS with comprehensive population-based cohort data. Such design has the potential to not only minimize the impact of confounding bias and reverse causation but also discover intermediate phenotypes or mediators for the disease causal pathways. Given the vital role of autoimmunity and inflammation in both AIDs and tumorigenesis, we hypothesize that peripheral immune and inflammatory biomarkers may serve as potential mediators that bridge AIDs and cancer development^26,27^. Here, we comprehensively investigated the associations between AIDs, genetic predispositions (i.e., disease-specific and combined AID-PRSs), and subsequent risk of overall cancer and a broad range of individual cancers.

## Methods

### Study design

**Figure 1:**
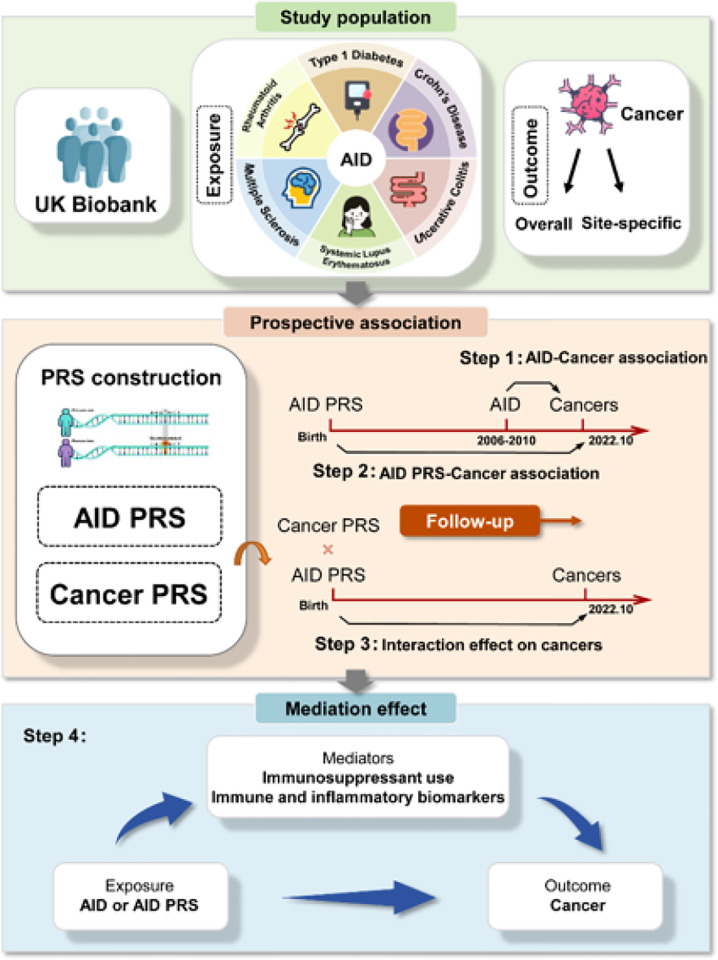
Flowchart of the study design. AID, autoimmune disease; PRS, polygenic risk score.

The flowchart shows the overall study design (Fig. 1). In brief, we initially evaluated the associations between prevalent AIDs at baseline and subsequent risk of cancer. We then constructed disease-specific and combined PRSs (both sexes and sex-specific) for six common AIDs and assessed the relationship between genetic predisposition to AIDs and cancer. Linkage disequilibrium (LD) score regression was employed to evaluate their genetic correlations. Interaction analysis between AID-PRS and cancer PRS was subsequently performed. Finally, we estimated the mediation effect of peripheral immune and inflammatory biomarkers and immunosuppressant use on AIDs at baseline or genetic predisposition to AIDs and cancer.

### Study population

The UK Biobank (UKBB), established between 2006 and 2010, is a large population-based prospective cohort comprising over 500,000 adults from 22 centers in the United Kingdom. Details on the recruitment, cohort design, and data collection of UKBB have been described in a previous publication^28^. The ethical review of UKBB has been approved by the North West Multicenter Research Ethical Committee (11/NW/0382). All participants provided written informed consent.

After excluding participants with missing genotype data, sex mismatch (difference between self-reported and genetically inferred sex), nonmelanoma skin cancer, non-European ancestry, and individuals with kinship, a total of 356,339 individuals (68,008 cancer cases and 288,331 controls) aged 37–74 years remained (Figure S1). Details on the identification of individuals with kinship are described in Supplementary Methods. This study sample was used to estimate the relationship between genetic predisposition to AIDs and cancer (subset II). After further excluding participants with cancer at recruitment, a total of 334,749 individuals (46,418 incident cancer cases and 288,331 controls) remained, which was used to estimate the observational associations between baseline AIDs and incident cancer (subset I).

### Definition of outcome

The outcome was cancer diagnosis, which was defined as malignant neoplasms and coded using the International Classification of Diseases (ICD, ICD-9: 140–208 and ICD-10: C00-C97) in the UKBB through record electronic linkage with hospital data and cancer registry. This study ascertained overall cancer, 6 system categories (hematological cancer, digestive cancer, urinary cancer, female genital cancer, male genital cancer, and HPV-related cancer), and 23 site-specific cancers. Overall cancer covered all cancers except nonmelanoma skin cancer (ICD-9: 173, ICD-10: C44). Details on cancer definition and classification are shown in Table S1. The start of the follow-up period was defined as the date of enrolment in the UKBB for assessing the observational associations between AIDs at baseline and subsequent risk of incident cancer, whereas the date of birth was used for estimating the relationship between genetic predisposition to AIDs and cancer (including both incident and prevalent cases). The follow-up was censored at the date of cancer diagnosis, death, or date of final registry update (October 31, 2022), whichever occurred first.

### Assessment of autoimmune diseases

Six common AIDs at recruitment were considered as exposure of interest, including systemic lupus erythematosus (SLE), rheumatoid arthritis (RA), multiple sclerosis (MS), Crohn’s disease (CD), ulcerative colitis (UC), and T1D (ICD codes: 7100 and M32 for SLE, 7140 and M05 for RA, 340 and G35 for MS, 555 and K50 for CD, 556 and K51 for UC, E10 for T1D).

### Genotyping and imputation

In UKBB, genome-wide genotyping data were generated for 488,377 participants using two similar arrays, including the UK Biobank Axiom array covering 825,927 genetic markers for 438,427 individuals and UK BiLEVE Axiom Array covering 807,411 genetic markers for 49,950 individuals. The two arrays share 95% of single-nucleotide polymorphism (SNPs) tested. Imputation was carried out using IMPUTE2 software, with the merged UK10K and 1000 Genomes Phase 3 as reference panel. Details on genotyping process, DNA arrays, and quality control (QC) have been described elsewhere^29^.

### Construction of autoimmune disease polygenic risk scores

For each AID, risk-associated variants with genome-wide significance (*P* < 5×10^− 8^) and corresponding effect sizes (beta) were obtained from published GWASs with the largest sample size (Table S2) by systematically searching the AID GWAS of European ancestry in PubMed and GWAS Catalog (https://www.ebi.ac.uk/gwas/). The LD clumping (r^2^ < 0.1 in ± 500 kb window) was performed to obtain independent susceptibility variants. We then constructed the PRS using independent variants for each AID following an additive model^30^. The details of the method are described in Supplementary Methods. We further combined individual AID PRS into a composite PRS (CAID-PRS), weighted by incidence^30^, as follows:

$${\text{CAID}-\text{PRS}=\sum }_{n=1}^{n}h_{n}{\text{PRS}}_{i,n}$$,

where h_n_ is the age-standardized incidence of individual AID_n_ among the UK population^31–33^ (Table S3) and PRS_i,n_ is the corresponding PRS of individual AID_n_ for the i^th^ participant in the UKBB. For sex-specific CAID-PRS, we used the sex-specific individual AID-PRS and corresponding sex-specific disease incidence in UK accordingly. Details on SNPs used to construct AID-PRSs (both sexes and sex-specific) are shown in Tables S4-S9.

### Construction of cancer polygenic risk scores

For the 8 significant AID-related cancer types, we also constructed corresponding cancer PRS using the same approach that was used to develop individual AID-PRS (Table S10). Given the small sample size of GWAS for anal cancer and oral cavity cancer, we combined existing GWAS data using the METAL software^34^ for both cancers. Independent SNPs with *P* < 1×10^− 6^ for anal cancer were used to construct anal cancer PRS. SNPs used to derive individual cancer PRSs are shown in Table S11.

### Immune and inflammatory biomarker assays

Blood samples were collected at enrolment and typically analyzed at the UKBB central laboratory within 24 h of blood draw. Blood count, including 13 blood cell counts or percent, was measured using the quantitative and automated Beckman Coulter LH750 haematology analyzer. C-reactive protein (CRP) was measured through Immuno-turbidimetric methodology using the Beckman Coulter AU5800 analytical platform. Details on sample selection, measurement, processing, and QC have been described elsewhere (https://biobank.ndph.ox.ac.uk/ukb/refer.cgi?id=1453). The missing measurements of blood count and CRP were imputed by median values. The units of measurement for blood cell counts and CRP are shown in Table S12.

## Statistical analysis

### Assessment of associations between prevalent AIDs and incident cancer

Cox proportional hazards (CPH) models were employed to assess the association between prevalent AIDs at baseline (yes or no) and the risk of incident cancer and estimate hazard ratios (HRs) and 95% confidence intervals (CIs). The Schoenfeld residuals method was used to assess the proportional hazard assumption. Model was adjusted for age at recruitment, sex, family history of cancer, education level, oily fish consumption, processed meat, aspirin use, vitamin supplement, physical activity, Townsend deprivation index (TDI), body mass index (BMI), smoking, and alcohol drinking (model i). Definitions of these covariates are shown in Table S13. For missing data, continuous variables were imputed with median values, while categorical variables were replaced with a missing indicator. Stratified analysis by sex was also performed.

### Assessment of associations between genetic predisposition to AIDs and cancer

First, we evaluated the association of CAID-PRS and individual AID-PRSs with the risk of AIDs using a logistic regression model in the whole population. Model was adjusted for age at recruitment, sex, assessment center, and first 10 genetic principal components (PCs). CPH models were employed to assess the association between AID-PRSs (combined and disease-specific) and the risk of cancer and estimate HRs and 95% CIs. Model was adjusted for age at recruitment, sex, assessment center, and first 10 genetic PCs (model 1). When appropriate, we further assessed each sex-specific PRS within the corresponding sex subgroup for the analyses described above.

In addition, we estimate overall genetic correlations between AIDs and cancer based on GWAS summary statistics. The information on GWAS summary statistics for AIDs and cancer is presented in Tables S2 and S10. Details for this analysis are described in Supplementary Methods.

### Estimation of interaction between AID-PRS and cancer-PRS on cancer risk

The association between individual cancer PRSs and the corresponding cancer risk was estimated using a logistic regression model in the whole population or sex-specific population (for sex-specific cancer). Model was adjusted for age at recruitment, sex, assessment center, and first 10 genetic PCs. Then, we evaluated whether there was a significant interaction between binary AID-PRS (binary: low < median value, high ≥ median value, based on its population distribution) and binary cancer-PRS on cancer risk by adding multiplicative interaction term in above-mentioned multivariable-adjusted CPH model 1. For significant interaction pairs, we further performed stratified analysis by the binary cancer-PRS to estimate the association between the corresponding AIDPRS and cancer risk. Heterogeneity across the binary cancer-PRS was tested to determine whether the AID-PRS-cancer association differed in low and high cancer-PRS groups.

### Mediation analysis of biomarkers and immunosuppressant use on AIDs, AID-PRSs and cancer

We performed a mediation analysis to assess whether peripheral immune and inflammatory biomarkers or immunosuppressant use contribute to the associations between genetic predisposition to AIDs and cancer. The R package ‘mediation’^35^ was utilized to perform the mediation analysis and estimated mediation proportion. First, presumed mediators, including immunosuppressant use and immune (i.e., 13 blood cell count or percent) and inflammatory biomarkers (i.e., CRP), were regressed by AID-PRS in multivariable linear or logistic regression models. AID-PRS-related biomarkers or immunosuppressant use were subsequently evaluated for their associations with incident cancer in CPH models. Finally, cancer incidence was regressed by AID-PRS, biomarkers or immunosuppressant use (mediator), and covariates by fitting the parametric survival regression model. Similarly, we also evaluated the mediation effect of biomarkers and immunosuppressant use on the relationships between baseline AIDs and cancer. The same covariates indicated in the model 1 or i were adjusted when appropriate.

### Sensitivity analysis

To assess the robustness of the findings, we further performed several sensitivity analyses. Details on sensitivity analyses are described in Supplementary Methods. All data cleaning and statistical analyses were conducted in R version 4.2.2, unless otherwise specified.

## Results

### Baseline characteristics

Table S14 summarizes the baseline characteristics of participants in both the observational study (subset I) and genetic study (subset II). In subset I, over a median follow-up time of 13.59 (IQR: 12.70, 14.33) years (started from date of recruitment), 46,418 incident cancer cases were documented among 334,749 participants. In subset II of 356,339 participants, during a median follow-up time of 69 (IQR: 62, 76) years (started from birth date), 68,008 individuals were diagnosed with cancer, a similar distribution of characteristics was observed between the two subsets.

### Associations between AIDs at baseline and cancer

**Figure 2:**
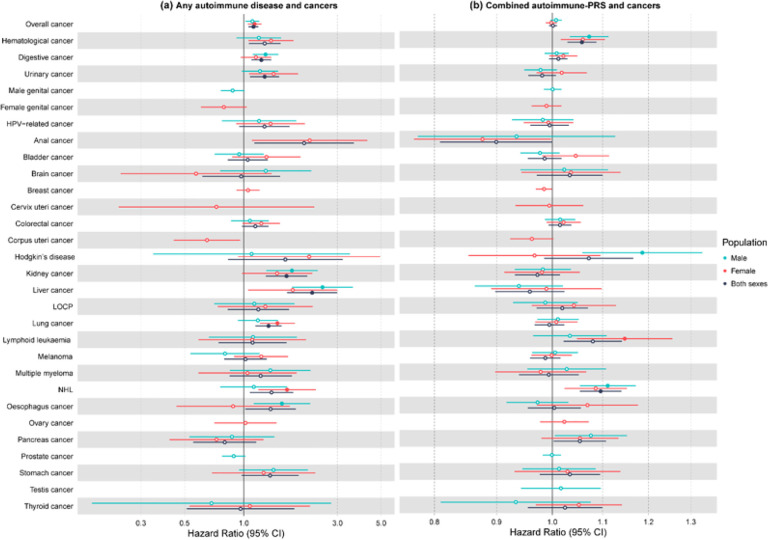
Forest plots showing (a) the associations between any autoimmune disease at baseline (yes or no) and cancer; (b) combined autoimmune disease polygenic risk score (PRS) (per SD increase) and cancer in the UK Biobank. Only association for those with at least 100 events is shown in this plot. Solid circle indicates significant association after multiple testing correction (false discovery rate-adjusted P<0.05). NHL, non-Hodgkin’s lymphoma; LOCP, malignant neoplasms of lip, oral cavity and pharynx.

Compared to those without AID at baseline, participants with any pre-existing AID were at a significantly higher risk of developing overall cancer (HR: 1.12, 95% CI: 1.06–1.19, FDR-adjusted *P* = 1.75×10^− 3^), liver (HR: 2.23, 95% CI: 1.66–2.99, FDR-adjusted *P* = 6.51×10^− 6^), kidney (HR: 1.65, 95% CI: 1.30–2.10, FDR-adjusted *P* = 1.26×10^− 3^), lung (HR: 1.34, 95% CI: 1.15–1.56, FDR-adjusted *P* = 2.46×10^− 3^), digestive (HR: 1.23, 95% CI: 1.10–1.38, FDR-adjusted *P* = 3.89×10^− 3^), and urinary cancers (HR: 1.28, 95% CI: 1.08–1.51, FDR-adjusted *P* = 0.03) (Fig. 2a, Table S15). Firth’s bias-reduced logistic regression confirmed these findings, with similar effect sizes and statistical significance. These associations were slightly attenuated after further adjustment for immunosuppressant use (Table S16). In sex stratified analysis, AID was associated with a higher risk of digestive, kidney, liver, and esophageal cancers in males and showed increased risks for overall cancer, lung cancer, and non-Hodgkin’s lymphoma (NHL) in females (Table S17).

### Associations between genetic predisposition to AIDs and AID incidence

Three distinct CAID-PRSs (both sexes and sex-specific) and 18 individual AID-PRSs (both sexes and sex-specific) for six AIDs were constructed. CAID-PRS was significantly associated with increased risk of CD, RA, SLE, T1D, and UC in the whole population and sex-specific populations except for SLE in males (Table S18). However, CAID-PRS was inversely associated with MS risk in the whole population and males. For individual AID-PRSs, all of them exhibited strongly significant associations with increased risk of the corresponding disease in the whole population and sex-specific populations (Table S19). Furthermore, we observed an inverse association between RA-PRS and MS risk.

### Relationships of genetic predisposition to AIDs with cancer

We further estimated the association of genetic predisposition to AIDs with cancer and found that CAID-PRS, reflecting overall genetic predisposition to AIDs, was significantly associated with increased risk of hematological cancer (HR [95% CI] per SD increase: 1.06 [1.03–1.09]) and NHL (HR [95% CI] per SD increase: 1.10 [1.05–1.14]) in the whole population (Fig. 2b, Table S20). Similar effect sizes and statistical significance were observed using Firth’s logistic regression. In the sex-stratified analysis, CAID-PRS was associated with an increased risk of hematological cancer, Hodgkin’s disease, and NHL in males and lymphoid leukemia in females (Fig. 2b, Table S21).

**Figure 3:**
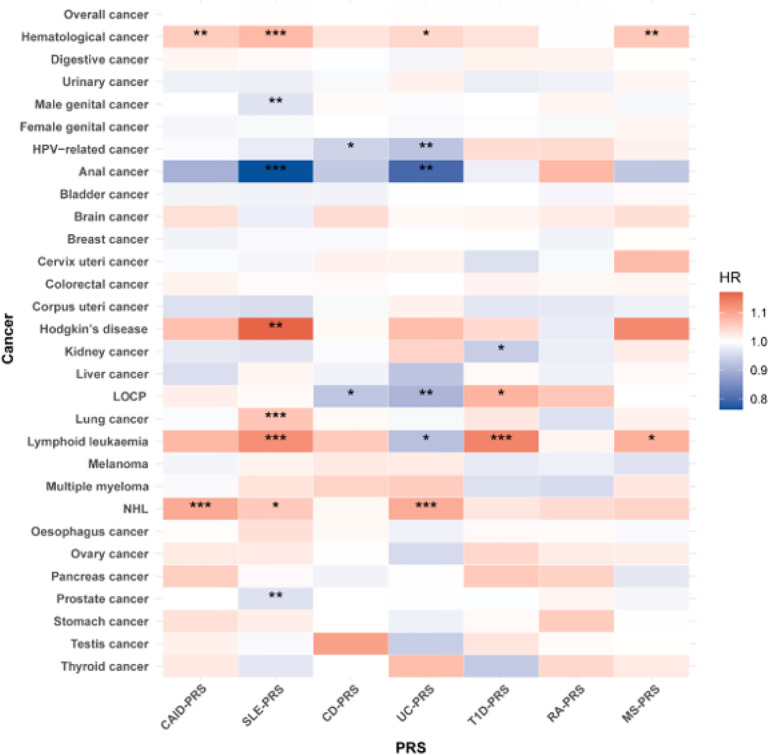
Heatmap showing associations between autoimmune disease polygenic risk scores (PRSs) (disease-specific and combined) (per SD increase) and cancer risk. *, false discovery rate (FDR)-adjusted *P*<0.05. **, FDR-adjusted *P*<0.01. ***, FDR-adjusted *P*<0.001. NHL, non-Hodgkin’s lymphoma; LOCP, malignant neoplasms of lip, oral cavity and pharynx; CAID, combined autoimmune disease; CD, Crohn’s disease; MS, multiple sclerosis; RA, rheumatoid arthritis; SLE, systemic lupus erythematosus; T1D, type 1 diabetes mellitus; UC, ulcerative colitis; HR, hazard ratio.

For individual AID-PRSs, we found 21 significant associations covering 5 PRSs and 11 cancers and 15 additional associations in sex-stratified populations (FDR-adjusted *P* < 0.05) (Fig. 3). Of them, SLE-, UC-, and T1D-PRS showed complex cross-cancer effects. Specifically, per SD increase in SLE-PRS was associated with a higher risk of Hodgkin’s disease (HR [95%CI]: 1.17 [1.08–1.27]), NHL (HR [95%CI]: 1.06 [1.02–1.10]), lymphoid leukemia (HR [95%CI]: 1.13 [1.07–1.19]), and lung cancer (HR [95%CI]: 1.06 [1.04–1.09]), whereas it was associated with a decreased risk of anal (HR [95%CI]: 0.76 [0.68–0.86]) and prostate cancers (HR: 0.96, 95%CI: 0.95–0.98) (Table 1, Fig. 3, Table S21). UC-PRS (per SD increase) was associated with an increased risk of NHL (HR [95%CI]: 1.09 [1.05–1.14]) and a decreased risk of anal cancer (HR [95%CI]: 0.80 [0.72–0.89]), malignant neoplasms of lip, oral cavity and pharynx (LOCP) (HR [95%CI]: 0.91 [0.86–0.95]), lymphoid leukemia (HR [95%CI]: 0.92 [0.87–0.97]) and HPV-related cancer (HR [95%CI]: 0.93 [0.89–0.96]). A similar CD-PRSHPV-related cancer association was found (HR [95%CI]: 0.94 [0.91–0.98]). T1D-PRS (per SD increase) showed an increased risk of LOCP (HR [95%CI]: 1.08 [1.03–1.14]) and lymphoid leukemia (HR [95%CI]: 1.14 [1.08–1.20]) but a decreased risk for kidney cancer (HR [95%CI]: 0.94 [0.90–0.98]). Immunosuppressant use had a minimal impact on these associations (model 2) (Table 1). Excluding individuals who reported immunosuppressant use also had little impact (data not shown). LDSC regression did not show any significant genetic correlations between AIDs and cancer (Table S22).

### Interaction effect between AID-PRS and cancer PRS on cancer

To determine whether the effects of AID-PRSs are independent of genetic predisposition to cancer, we further constructed and included individual cancer PRSs in the model. These cancer PRSs were significantly associated with an increased risk of their corresponding cancer, except for anal cancer and LOCP, probably due to insufficient power (Table S23). The associations between individual AID-PRSs and cancer remained significant or nominally significant with consistent effect direction except for SLE-PRS with lung cancer and NHL, UC-PRS with lymphoid leukemia, and SLE-PRS with prostate cancer when the corresponding cancer PRS was adjusted (Table 1). In the subsequent interaction analysis, a significant interaction effect between SLE-PRS and prostate cancer-PRS on prostate cancer risk was observed (*P*_interaction_=0.03) (Table 1). When stratified by prostate cancer-PRS (low, high), SLE-PRS showed borderline significant associations in both low and high prostate cancer-PRS populations with opposite association directions (*P*_heterogeneity_=0.03) (Table S24).

### Associations between AIDs at baseline and cancer with further adjustment

We further assessed the association between individual AID and cancer risk by restricting to the significant AID-PRS and cancer pairs. In multivariate-adjusted model i, ii, and iii, consistent with the association of AID-PRS with cancer, any AID and SLE at baseline were significantly associated with a higher risk of NHL. On the contrary, SLE and UC at baseline were positively associated with anal cancer. After additionally adjusting for immunosuppressant use (model iv), these associations became non-significant for most cancer types, implicating that these observational associations were more likely to be confounded by immunosuppressant use (Table S25). The association between UC and anal cancer remains significant in this analysis.

### Mediating role of immune-related and inflammatory biomarkers in AIDs and cancer

We explored potential mediators that may contribute to the associations between pre-existing AIDs or genetic predisposition to AIDs and cancer. AID at baseline was significantly associated with levels of 11 biomarkers (Table S26) and the use of immunosuppressant (FDR-adjusted *P* < 0.05) (Table S27). A total of 63 associations between these AID-related biomarkers and various cancer types were found (FDR-adjusted *P* < 0.05) (Table S28). Immunosuppressant use was associated with a higher risk of overall cancer, hematological cancer, urinary cancer, and 6 individual cancers (Table S29). Mediation analyses revealed immunosuppressant use and 9 biomarkers (i.e., CRP, basophil, high light scatter reticulocyte [HLSR], monocyte, neutrophil, platelet, red blood cell [RBC], RBC distribution width, white blood cell [WBC]) partly mediated the associations of baseline AID with the risk of overall cancer, urinary cancer, digestive cancer, and 3 individual cancers (Table S27). The mediated proportion ranged from 14.84–72.79%, of which immunosuppressant use accounted for 5.10–22.82%. For AID-PRSs, after multiple-testing correction, 54 associations were found significant between AID-PRSs and peripheral biomarkers, covering 6 AID-PRSs and 13 blood counts and CRP (FDR-adjusted *P* < 0.05) (Table S30). CAID-, T1D-, and MS-PRSs were positively related to immunosuppressant use (FDR-adjusted *P* < 0.05) (Table S31). 59 significant associations regarding AID-PRS-related biomarkers and various cancer types were observed (Table S32). Finally, mediation analyses revealed 8 biomarkers (i.e., CRP, RBC, RBC distribution width, WBC, platelet, basophil, neutrophil, and nucleated red blood cell percentage) served as mediators in the relationship between CAID-PRS and 5 individual AID-PRSs and the risk of hematological cancer, male genital cancer, and 6 individual cancers, accounting for 0.57–16.96% of these associations (Table 2). On the other hand, the majority of the associations between AID-PRSs and cancer were not explained by immunosuppressant use, except for the associations of CAID-PRS with NHL and hematological cancer and MS-PRS with hematological cancer, with the mediated proportion of immunosuppressant use ranging from 0.62–3.84%. As an example, CAID-PRS was positively associated with NHL risk, partly mediated by increased levels of peripheral CRP, RBC distribution width, and WBC counts, as well as lowered RBC counts and the use of immunosuppressant, with a total mediation proportion of 10.32%.

## Discussion

In this large population-based cohort study, we systematically investigated the relationship between pre-existing AIDs at baseline, genetic susceptibility to AIDs, and cancer risk. We found that participants with a higher predisposition to AIDs had an elevated risk of hematological cancers and several solid tumors. We also found that immunosuppressant use, alteration of blood cell counts, and CRP are important mediators for the majority of the observed associations between AIDs and cancer. However, only blood cell counts and CRP partly mediated the associations of AID genetic predisposition with cancer, while overlapping susceptibility between the two diseases or the use of immunosuppressant drugs were generally not found as potential mediators. Therefore, our study provides important evidence that, rather than immunosuppression caused by treatment or other medical conditions, additional mechanisms may contribute to carcinogenesis in individuals with an elevated predisposition to AIDs.

### Hematological cancer

The link between AID and hematological cancer was most studied among all cancer types. Prior studies have shown an increased risk of hematological cancer in AID patients, especially among SLE patients, and a higher risk of NHL in RA patients^18,36^. Our results confirm these findings although the observed associations for any baseline AID did not pass the stringent multiple testing correction threshold. In line with the non-genetic associations, we also found consistent significant (or suggestive) positive relationship between genetic predisposition to all included AIDs and hematological cancer risk except for RA. This was mainly because only a nominally significant association was found for genetic predisposition to RA with increased risk of NHL but not with other hematological cancer types. Several possible mechanisms have been speculated, including persistent chronic inflammation and overstimulation of B cells coupled with other defects in the immune system^37,38^. It has been hypothesized that hyperactivity of B cells results in secretion of autoantibodies and production of proinflammatory cytokines, eventually leading to immune cell dysregulation, chronic inflammation, and ultimately cancer.

### Solid tumors

AID is also thought to be closely related to solid tumors. In the present study, we found a strong association between AID and anal cancer. Anal cancer is one of the most rapidly increasing cancer types particularly among older adult women^39^. A higher rate is found in immune compromised population such as human immunodeficiency virus (HIV) patients and those who use immunosuppressant drugs to treat AIDs or prevent transplant rejection^13,40,41^. Infection by the human papillomavirus (HPV) and homosexual activities (e.g., men who have sex with men) are two important risk factors for the malignancy^41^. The results of our study did not support a shared genetic susceptibility between AIDs and anal cancer, i.e., individuals who are genetically prone to AIDs (SLE and UC) do not have a higher risk of anal cancer; instead, a robust inverse association was found. The exact mechanism remains to be explored but an enhanced immunity to reduce HPV infections is a possible explanation as significant inverse associations were also observed for UC- and CD-PRS and HPV-related cancers. Although we did not adjust or exclude individuals living with HIV infection from our study population, it is not a particular concern since prior literature indicated an extremely small prevalence in this population^42^. The association was more pronounced in female again supported that HIV infection and men who have sex with men are unlikely to be critical confounders^39,43^. Additional datasets with large sample sizes and functional data are needed to validate our findings and elucidate underlying biological mechanisms.

Lung cancer is more common in SLE patients compared to the general population while the risk of prostate cancer is lower in male patients^44–47^. Smoking as the most contributing risk factor for lung cancer is also found prevalent among male SLE patients. Compared with nonsmokers, the lung cancer risk increased nearly four-fold among SLE patients who smoked^46^. We also identified a positive association of SLE-PRS with lung cancer risk in this study. Of note, the association diminished to null when adjusting for lung cancer PRS. As it has been previously shown that the effect of smoking on lung cancer risk doubles in those at high genetic risk of the malignancy^48^, our findings highlight the benefits of smoking cessation and early screening of lung cancer in smokers who are genetically susceptible to SLE. Prior studies provided a complex relationship between different AIDs and prostate cancer risk.

A positive association with prostate cancer was reported for ulcerative colitis while an inverse relationship was found for RA and SLE^10,46,49^. However, another study using data from US military veterans did not support a causal link between RA and prostate cancer, although the generalizability of the study findings to other populations is unclear^50^. A reduced level of testosterone/altered hormone activity is suggested to contribute to the lower risk of prostate cancer in SLE patients^51^ and aberrant fecal microbiome may also play a role^52^. The interaction identified for prostate cancer-PRS and SLE-PRS was novel. In the stratified analysis, the inverse association of SLE-PRS appeared to be driven by individuals who also had a relatively low susceptibility to prostate cancer (< median). It is possible that the protective effect of SLE-PRS is offset by the stronger genetic risk factor, i.e., prostate cancer PRS in the other half of the group.

### Mediators

Our findings expanded the evidence regarding how AIDs or genetic susceptibility to AIDs contributed to cancer initiation or progress. Immunosuppressant use, alteration of peripheral blood counts, and CRP were identified as likely mediators of the associations between AIDs at baseline and cancer. For instance, the use of immunosuppressant mediated 16.59% of the association between AIDs at baseline and overall cancer risk, and 56.20% mediated by 8 peripheral biomarkers (e.g., basophil, CRP, high light scatter reticulocyte, monocyte, neutrophil, platelet, RBC width, WBC).

Immunosuppressive treatments and altered intrinsic immune functions may act as likely mediators linking AID to impaired immune function, and subsequently, an increased risk of cancer. Similar to our findings, previous studies have reported increased cancer risk associated with the use of immunosuppressants in patients with RA or inflammatory bowel disease^53,54^. As an important part of immune system, white blood cells circulate in blood and respond to injury or illness, and have been found to be closely related to not only hematological cancer but also solid tumors^55,56^. Red blood cells were previously thought to be immunologically inert. Recently, however, the role of red blood cells in the immune system has been emphasized. Red blood cells are considered immune sentinels and dynamic reservoirs of cytokines. They have been shown to influence immune function and can induce inflammatory responses following transfusion^57,58^. Notably, the majority of the associations between genetic susceptibility to AIDs and cancer were independent of the use of immunosuppressant drugs but partly mediated via peripheral red blood counts, platelet, white blood counts, and CRP. These findings support that altered peripheral immune and inflammatory biomarkers involve in the etiological pathway between AIDs and cancer in addition to immunosuppression caused by treatment, which informs new opportunities for target cancer prevention and treatment.

### Strengths and limitations

The study has the advantage of a prospective design, large sample size, and long follow-up. The high-quality genetic and epidemiological data available in the UKBB allowed us to comprehensively examine the role of AIDs in cancer development and tease apart the contributions of genetic factors, medication use, and potential molecular mediators. Furthermore, the robust associations observed in the mediation analysis suggest that the identified mediation effects are unlikely to be due to chance or significantly affected by bias.

Our study also has a few limitations. For example, the sample sizes for several rare cancers were limited; therefore, the association estimates may be imprecise or the power to detect an association may have been insufficient. Similarly, evaluating the mediators between individual AID at baseline and rare cancers is challenging due to the small number of events. In addition, proteomics and metabolomics data were only available in about one tenth of the study population, limiting our ability to perform a more comprehensive mediation analysis. A revisit to this problem could provide new insights when additional data become available. Finally, the generalizability of our findings is limited by the predominance of participants of European ancestry enrolled in the UKBB. The relationship between genetic predisposition to AIDs and cancer warrants investigation in other racial and ethnic groups.

## Conclusions

This study demonstrated that genetic predisposition to AIDs was associated with hematological cancer and several solid tumors. The observed associations were generally independent of inherited genetic susceptibility to cancer or immunosuppression resulting from immunosuppressive treatment; rather, they may be partly mediated via alteration of peripheral immune and inflammatory biomarkers. Our study provides new evidence linking AIDs to cancer and supports the concept that targeted screening and cancer prevention might be beneficial in potentially vulnerable populations.

## Figures and Tables

**Table 1:** The associations of autoimmune diseases polygenic risk scores (PRSs) (per SD increase) with cancer risk (FDR-adjusted *P* < 0.05) in the UK Biobank.

**Exposure**	**Outcome**	**Population**	**Case**	**Control**	**Model 1_**			**Model 2**		**Model 3**		Interaction (Autoimmune-PRS*Cancer-PRS) ^a^	
					**HR (95%CI)**	**P**	**FDR-adjusted P**	**HR (95%CI)**	**P**	**HR (95%CI)**	**P**	**HR (95% CI)**	**P**_**interaction**_
SLE-PRS	Anal cancer	Both sexes	337	356002	0.76 (0.68–0.86)	6.87×10^− 6^	6.20×10^− 4^	0.76 (0.68–0.86)	6.73×10^− 6^	0.76 (0.68–0.86)	6.60×10^− 6^	1.22 (0.67–2.23)	0.51
UC-PRS	Anal cancer	Both sexes	337	356002	0.80 (0.72–0.89)	6.73×10^− 5^	2.05E×10^− 3^	0.80 (0.72–0.89)	6.82×10^− 5^	0.80 (0.72–0.89)	6.69×10^− 5^	0.66 (0.35–1.22)	0.18
SLE-PRS	Hodgkin’s disease	Both sexes	533	355806	1.17 (1.08–1.27)	9.87×10^− 5^	2.62×10^− 3^	1.17 (1.08–1.27)	1.02×10^− 4^	1.12 (1.03–1.22)	7.34×10^− 3^	0.73 (0.43–1.25)	0.25
T1D-PRS	Kidney cancer	Both sexes	2094	354245	0.94 (0.90–0.98)	3.06×10^− 3^	4.06×10^− 2^	0.94 (0.90–0.98)	2.46×10^− 3^	0.94 (0.90–0.98)	2.50×10^− 3^	0.98 (0.82–1.16)	0.80
CD-PRS	LOCP	Both sexes	1603	354736	0.93 (0.88–0.97)	2.58×10^− 3^	3.80×10^− 2^	0.93 (0.88–0.97)	2.52×10^− 3^	0.93 (0.88–0.97)	1.96×10^− 3^	1.01 (0.83–1.23)	0.90
T1D-PRS	LOCP	Both sexes	1603	354736	1.08 (1.03–1.14)	8.93×10^− 4^	1.65×10^− 2^	1.08 (1.03–1.14)	1.05×10^− 3^	1.08 (1.03–1.13)	2.38×10^− 3^	1.12 (0.92–1.36)	0.26
UC-PRS	LOCP	Both sexes	1603	354736	0.91 (0.86–0.95)	9.99×10^− 5^	2.62×10^− 3^	0.91 (0.86–0.95)	9.72×10^− 5^	0.91 (0.86–0.95)	1.09×10^− 4^	0.99 (0.81–1.20)	0.90
SLE-PRS	Lung cancer	Both sexes	4822	351517	1.06 (1.04–1.09)	8.94×10^− 6^	6.62×10^− 4^	1.06 (1.04–1.09)	9.25×10^− 6^	0.98 (0.96–1.01)	2.61×10^− 1^	1.00 (0.89–1.12)	0.96
MS-PRS	Lymphoid leukemia	Both sexes	1245	355094	1.09 (1.03–1.15)	2.64×10^− 3^	3.80×10^− 2^	1.09 (1.03–1.15)	2.72×10^− 3^	1.08 (1.02–1.14)	6.49×10^− 3^	1.20 (0.94–1.52)	0.14
SLE-PRS	Lymphoid leukemia	Both sexes	1245	355094	1.13 (1.07–1.19)	1.20×10^− 5^	7.77×10^− 4^	1.13 (1.07–1.19)	1.22×10^− 5^	1.09 (1.04–1.15)	9.27×10^− 4^	1.14 (0.90–1.45)	0.28
UC-PRS	Lymphoid leukemia	Both sexes	1245	355094	0.92 (0.87–0.97)	3.65×10^− 3^	4.61×10^− 2^	0.92 (0.87–0.97)	3.62×10^− 3^	0.95 (0.89–1.00)	5.23×10^− 2^	1.00 (0.79–1.27)	1.00
T1D-PRS	Lymphoid leukemia	Both sexes	1245	355094	1.14 (1.08–1.20)	2.25×10^− 6^	5.54×10^− 4^	1.14 (1.08–1.20)	2.83×10^− 6^	1.07 (1.01–1.13)	1.62×10^− 2^	1.06 (0.84–1.35)	0.62
CAID-PRS	Non-Hodgkin’s lymphoma	Both sexes	2432	353907	1.10 (1.05–1.14)	5.40×10^− 6^	6.20×10^− 4^	1.09 (1.05–1.13)	1.63×10^− 5^	1.02 (0.98–1.07)	2.91×10^− 1^	1.13 (0.96–1.34)	0.15
SLE-PRS	Non-Hodgkin’s lymphoma	Both sexes	2432	353907	1.06 (1.02–1.10)	2.95×10^− 3^	4.02×10^− 2^	1.06 (1.02–1.10)	3.05×10^− 3^	0.96 (0.92–1.01)	9.70×10^− 2^	1.00 (0.85–1.18)	0.99
UC-PRS	Non-Hodgkin’s lymphoma	Both sexes	2432	353907	1.09 (1.05–1.14)	7.18×10^− 6^	6.20×10^− 4^	1.09 (1.05–1.14)	7.35×10^− 6^	1.10 (1.06–1.15)	1.68×10^− 6^	1.14 (0.97–1.33)	0.12
SLE-PRS	Prostate cancer	Male	12954	149093	0.96 (0.95–0.98)	1.77×10^− 5^	1.02×10^− 3^	0.96 (0.95–0.98)	1.76×10^− 5^	1.02 (1.00–1.04)	1.80×10^− 2^	1.08 (1.01, 1.17)	0.03
CAID-PRS	Hematological cancer	Both sexes	5023	351316	1.06 (1.03–1.09)	6.29×10^− 5^	2.04×10^− 3^	1.05 (1.03–1.08)	1.61×10^− 4^	-	-	-	-
MS-PRS	Hematological cancer	Both sexes	5023	351316	1.06 (1.03–1.09)	2.17×10^− 5^	1.02×10^− 3^	1.06 (1.03–1.09)	2.49×10^− 5^	-	-	-	-
SLE-PRS	Hematological cancer	Both sexes	5023	351316	1.08 (1.05–1.11)	2.94×10^− 8^	1.52×10^− 5^	1.08 (1.05–1.11)	3.11×10^− 8^	-	-	-	-
UC-PRS	Hematological cancer	Both sexes	5023	351316	1.05 (1.02–1.07)	1.67×10^− 3^	2.71×10^− 2^	1.05 (1.02–1.07)	1.72×10^− 3^	-	-	-	-
SLE-PRS	Male genital cancer	Male	13584	148463	0.96 (0.95–0.98)	2.57×10^− 5^	1.02×10^− 3^	0.96 (0.95–0.98)	2.55×10^− 5^	-	-	-	-
CD-PRS	HPV-related cancer	Both sexes	2847	353492	0.94 (0.91–0.98)	2.42×10^− 3^	3.69×10^− 2^	0.94 (0.91–0.98)	2.37×10^− 3^	-	-	-	-
UC-PRS	HPV-related cancer	Both sexes	2847	353492	0.93 (0.89–0.96)	4.38×10^− 5^	1.62×10^− 3^	0.93 (0.89–0.96)	4.38×10^− 5^	-	-	-	-

Results were derived from Cox proportional hazards regression analysis, with follow-up time defined as the date of birth to the date of incident outcome.

Model 1 adjusted for age at recruitment, sex (only adjusted in both sexes), assessment center, and first 10 genetic principal components (PCs).

Model 2 adjusted for model 1 + immunosuppressant use.

Model 3 adjusted for model 2 + the corresponding cancer PRS.

a Estimates of interaction effect were derived based on corresponding autoimmune-PRS (low: <median, high: >median) and cancer-PRS (low: <median, high: >median).

CAID, combined autoimmune disease; CD, Crohn’s disease; MS, multiple sclerosis; SLE, systemic lupus erythematosus; T1D, type 1 diabetes mellitus; UC, ulcerative colitis; LOCP, malignant neoplasms of lip, oral cavity and pharynx; FDR, false discovery rate; PRS, polygenic risk score; HR, hazard ratio; CI, confidence interval.

**Table 2:** Mediation analysis of the association between autoimmune polygenic risk scores (PRSs) and cancer, with blood cell count, CRP, or immunosuppressant use as mediators.

**Exposure**	**Mediator**	**Outcome**	**Exposure (per SD increase)-Outcome**		**Exposure (per SD increase)-Mediator**		**Mediator (per SD increase)-Outcome**		**Mediation effect**		
			**HR (95% CI)** ^a^	**FDR-adjusted *P*** ^a^	**Beta or OR (95% CI)** ^b^	**FDR-adjusted *P*** ^b^	**HR (95% CI)** ^c^	***P*** ^c^	**Mediated proportion (%)** ^d^	***P*** ^d^	**Total mediated proportion (%)**
CAID-PRS	CRP	NHL	1.10 (1.05–1.14)	6.20×10^− 4^	0.0751 (0.0612, 0.0891)	3.10×10^− 25^	1.09 (1.05, 1.12)	2.95×10^− 7^	2.07	< 2×10^− 16^	10.32
	RBC				−0.0016 (−0.0027, −0.0005)	1.07×10^− 2^	0.80 (0.76, 0.84)	1.62×10^− 19^	3.35	< 2×10^− 16^	
	RBC.width				0.0178 (0.0148, 0.0209)	5.37×10^− 29^	1.15 (1.11, 1.19)	1.18×10^− 17^	1.21	0.02	
	WBC				0.0246 (0.0180, 0.0311)	1.18×10^− 12^	1.07 (1.05, 1.08)	2.64×10^− 15^	0.66	< 2×10^− 16^	
	immunosuppressant use				1.38 (1.33, 1.43)	4.82×10^− 69^	3.04 (2.28–4.06)	3.49E-14	3.03	< 2×10^− 16^	
CAID-PRS	RBC.width	Hematological cancer	1.06 (1.03–1.09)	2.04×10^− 3^	0.0178 (0.0148, 0.0209)	5.37×10^− 29^	1.14 (1.11–1.16)	2.33×10^− 25^	5.69	< 2×10^− 16^	18.99
	CRP				0.0751 (0.0612, 0.0891)	3.10×10^− 25^	1.05 (1.02–1.07)	9.26×10^− 4^	2.55	< 2×10^− 16^	
	WBC				0.0246 (0.0180, 0.0311)	1.18×10^− 12^	1.17 (1.16–1.18)	7.55×10^− 260^	4.85	< 2×10^− 16^	
	RBC				−0.0016 (−0.0027, −0.0005)	1.07×10^− 2^	0.80 (0.77–0.83)	2.69×10^− 36^	2.06	0.04	
	immunosuppressant use				1.38 (1.33, 1.43)	4.82×10^− 69^	2.19 (1.72–2.77)	1.05E-10	3.84	< 2×10^− 16^	
CD-PRS	Platelet	LOCP	0.93 (0.88–0.97)	3.80×10^− 2^	0.4267 (0.2403, 0.6131)	2.09×10^− 5^	0.90 (0.85, 0.96)	1.08×10^− 3^	0.57	0.02	0.57
MS-PRS	Basophil	Lymphoid leukemia	1.09 (1.03–1.15)	3.80×10^− 2^	0.0003 (0.0001, 0.0004)	4.24×10^− 3^	1.14 (1.12, 1.16)	2.38×10^− 50^	0.82	< 2×10^− 16^	2.30
	RBC.width				0.0058 (0.0027, 0.0089)	5.79×10^− 4^	1.14 (1.09, 1.20)	3.05×10^− 8^	1.48	< 2×10^− 16^	
MS-PRS	Lymphocyte	Hematological cancer	1.06 (1.03–1.09)	1.02×10^− 3^	0.0091 (0.0054, 0.0128)	4.32×10^− 6^	1.11 (1.10–1.11)	< 1×10^− 300^	0.77	< 2×10^− 16^	5.66
	RBC.width				0.0058 (0.0027, 0.0089)	5.79×10^− 4^	1.14 (1.11–1.16)	2.33×10^− 25^	1.73	< 2×10^− 16^	
	Basophill				0.0003 (0.0001, 0.0004)	4.24×10^− 3^	1.08 (1.06–1.10)	1.56×10^− 15^	0.69	< 2×10^− 16^	
	WBC				0.0089 (0.0023, 0.0155)	1.55×10^− 2^	1.17 (1.16–1.18)	7.55×10^− 260^	1.85	< 2×10^− 16^	
	immunosuppressant use				1.06 (1.03, 1.10)	1.99×10^− 3^	2.19 (1.72–2.77)	1.05E-10	0.62	0.04	
SLE-PRS	RBC	Hodgkin's disease	1.17 (1.08–1.27)	2.62×10^− 3^	−0.0064 (−0.0075, −0.0053)	2.07×10^− 27^	0.85 (0.74, 0.99)	3.73×10^− 2^	1.50	< 2×10^− 16^	3.25
	RBC.width				0.0183 (0.0152, 0.0214)	1.98×10^− 30^	1.12 (1.02, 1.24)	2.44×10^− 2^	1.75	< 2×10^− 16^	
SLE-PRS	RBC	Lung cancer	1.06 (1.04–1.09)	6.62×10^− 4^	−0.0064 (−0.0075, −0.0053)	2.07×10^− 27^	0.96 (0.93, 0.99)	1.42×10^− 2^	2.47	< 2×10^− 16^	7.33
	RBC.width				0.0183 (0.0152, 0.0214)	1.98×10^− 30^	1.09 (1.07, 1.12)	4.40×10^− 13^	4.86	< 2×10^− 16^	
SLE-PRS	Platelet	Lymphoid leukemia	1.13 (1.07–1.19)	7.77×10^− 4^	−0.6342 (−0.8201, −0.4482)	9.78×10^− 11^	0.81 (0.76, 0.87)	9.33×10^− 9^	3.40	< 2×10^− 16^	9.95
	RBC				−0.0064 (−0.0075, −0.0053)	2.07×10^− 27^	0.86 (0.80, 0.93)	5.74×10^− 5^	3.54	< 2×10^− 16^	
	RBC.width				0.0183 (0.0152, 0.0214)	1.98×10^− 30^	1.14 (1.09, 1.20)	3.05×10^− 8^	3.01	< 2×10^− 16^	
SLE-PRS	Platelet	NHL	1.06 (1.02–1.10)	4.02×10^− 2^	−0.6342 (−0.8201, −0.4482)	9.78×10^− 11^	0.89 (0.85, 0.94)	9.16×10^− 6^	3.04	< 2×10^− 16^	16.96
	RBC				−0.0064 (−0.0075, −0.0053)	2.07×10^− 27^	0.80 (0.76, 0.84)	1.62×10^− 19^	8.74	< 2×10^− 16^	
	RBC.width				0.0183 (0.0152, 0.0214)	1.98×10^− 30^	1.15 (1.11, 1.19)	1.18×10^− 17^	5.18	< 2×10^− 16^	
SLE-PRS	Platelet	Prostate cancer	0.96 (0.95–0.98)	1.02×10^− 3^	−0.6342 (−0.8201, −0.4482)	9.78×10^− 11^	1.05 (1.03, 1.07)	7.27×10^− 6^	1.09	< 2×10^− 16^	5.09
	RBC				−0.0064 (−0.0075, −0.0053)	2.07×10^− 27^	1.08 (1.06, 1.10)	2.92×10^− 12^	2.26	< 2×10^− 16^	
	RBC.width				0.0183 (0.0152, 0.0214)	1.98×10^− 30^	0.96 (0.94, 0.98)	1.27×10^− 4^	1.73	< 2×10^− 16^	
SLE-PRS	RBC	Hematological cancer	1.08 (1.05–1.11)	1.52×10^− 5^	−0.0064 (−0.0075, −0.0053)	2.07×10^− 27^	0.80 (0.77–0.83)	2.69×10^− 36^	5.91	< 2×10^− 16^	12.76
	RBC.width				0.0183 (0.0152, 0.0214)	1.98×10^− 30^	1.14 (1.11–1.16)	2.33×10^− 25^	4.48	< 2×10^− 16^	
	Platelet				−0.6342 (−0.8201, −0.4482)	9.78×10^− 11^	0.91 (0.88–0.94)	1.66×10^− 7^	2.27	< 2×10^− 16^	
	NRBC				0.0001 (0.0000, 0.0002)	2.45×10^− 2^	1.02 (1.00–1.03)	3.98×10^− 2^	0.10	0.02	
SLE-PRS	RBC	Male genital cancer	0.96 (0.95–0.98)	1.02×10^− 3^	−0.0064 (−0.0075, −0.0053)	2.07×10^− 27^	1.08 (1.06–1.10)	5.95×10^− 12^	1.68	< 2×10^− 16^	4.59
	Platelet				−0.6342 (−0.8201, −0.4482)	9.78×10^− 11^	1.05 (1.03–1.07)	6.59×10^− 6^	1.15	< 2×10^− 16^	
	RBC.width				0.0183 (0.0152, 0.0214)	1.98×10^− 30^	0.96 (0.94–0.98)	1.61×10^− 4^	1.76	< 2×10^− 16^	
T1D-PRS	RBC	LOCP	1.08 (1.03–1.14)	1.65×10^− 2^	−0.0038 (−0.0049, −0.0026)	2.62×10^− 10^	0.88 (0.82, 0.94)	1.43×10^− 4^	2.83	< 2×10^− 16^	2.83
T1D-PRS	Neutrophil	Lymphoid leukemia	1.14 (1.08–1.20)	5.54×10^− 4^	0.0054 (0.0008, 0.0099)	3.23×10^− 2^	1.11 (1.05, 1.17)	3.60×10^− 4^	0.31	< 2×10^− 16^	3.93
	RBC				−0.0038 (−0.0049, −0.0026)	2.62×10^− 10^	0.86 (0.80, 0.93)	5.74×10^− 5^	1.71	< 2×10^− 16^	
	RBC.width				0.0123 (0.0092, 0.0154)	1.68×10^− 14^	1.14 (1.09, 1.20)	3.05×10^− 8^	1.91	< 2×10^− 16^	
UC-PRS	Platelet	LOCP	0.91 (0.86–0.95)	2.62×10^− 3^	0.9568 (0.7692, 1.1445)	9.76×10^− 23^	0.90 (0.85, 0.96)	1.08×10^− 3^	1.03	0.02	1.03
UC-PRS	Basophil	Lymphoid leukemia	0.92 (0.87–0.97)	4.61×10^− 2^	−0.0002 (−0.0004, −0.0001)	1.26×10^− 2^	1.14 (1.12, 1.16)	2.38×10^− 50^	0.82	0.04	9.20
	Platelet				0.9568 (0.7692, 1.1445)	9.76×10^− 23^	0.81 (0.76, 0.87)	9.33×10^− 9^	8.38	0.02	
UC-PRS	CRP	NHL	1.09 (1.05–1.14)	6.20×10^− 4^	0.0194 (0.0054, 0.0335)	1.26×10^− 2^	1.09 (1.05, 1.12)	2.95×10^− 7^	0.63	< 2×10^− 16^	1.92
	RBC.width				0.0070 (0.0039, 0.0101)	3.05×10^− 5^	1.15 (1.11, 1.19)	1.18×10^− 17^	1.29	< 2×10^− 16^	
UC-PRS	RBC.width	Hematological cancer	1.05 (1.02–1.07)	2.71×10^− 2^	0.0070 (0.0039, 0.0101)	3.05×10^− 5^	1.14 (1.11–1.16)	2.33×10^− 25^	2.72	< 2×10^− 16^	3.56
	CRP				0.0194 (0.0054, 0.0335)	1.26×10^− 2^	1.05 (1.02–1.07)	9.26×10^− 4^	0.84	< 2×10^− 16^	

a Results were derived from Cox proportional hazards regression analysis, with follow-up time defined as the date of birth to the date of incident outcome. Model was adjusted for age at recruitment, sex (only adjusted in both sexes), assessment center, and first 10 genetic principal components (PCs).

b Results were derived from linear regression (beta) for blood cell count and CRP and logistic regression (OR) for immunosuppressant use (yes or no). Model was adjusted for age at recruitment, sex, family history of cancer, education level, oily fish, processed meat, aspirin, vitamin, physical activity, Townsend deprivation index, body mass index, smoking, and alcohol drinking.

c Results were derived from Cox proportional hazards regression analysis, with follow-up time as defined as the date of enrolment to the date of incident outcome. Model was adjusted for age at recruitment, sex (only adjusted in both sexes), family history of cancer, education level, oily fish, processed meat, aspirin, vitamin, physical activity, Townsend deprivation index, body mass index, smoking, and alcohol drinking.

d Results were derived from mediation analysis based on linear regression and parametric survival regression model, with follow-up time defined as the date of birth to the date of incident outcome. Model was adjusted the same list of covariates as detailed above. CAID, combined autoimmune disease; CD, Crohn’s disease; MS, multiple sclerosis; SLE, systemic lupus erythematosus; T1D, type 1 diabetes mellitus; UC, ulcerative colitis; CRP, C-reactive protein; RBC, red blood cell (erythrocyte); WBC, white blood cell (leukocyte); RBC.width, red blood cell (erythrocyte) distribution width; NRBC, nucleated red blood cell percentage; HR, hazard ratio; CI, confidence interval; OR, odds ratio; FDR, false discovery rate; NHL, Non-Hodgkin's lymphoma; LOCP, malignant neoplasms of lip, oral cavity and pharynx.

## Data Availability

The UKBB data are available under restricted access for data protocol, access can be obtained by registering and applying at http://ukbiobank.ac.uk/register-apply/. This study used the UKBB data under application numbers 66354 and 99685.
